# Supplementary material for: Using Genetics to Examine a General Liability to Childhood Psychopathology
Source: Behav Genet. 2019 Dec 11;50(4):213–20. doi: 10.1007/s10519-019-09985-4 (PMC7355267; doi:10.1007/s10519-019-09985-4)
Supplement: Supplementary file 1 — Supplementary material 1 (DOCX 454 kb) [file 10519_2019_9985_MOESM1_ESM.docx]

**Supplementary Material**

**Generating polygenic risk scores**

In total 9912 ALSPAC children were genotyped using the Illumina HumanHap500-quad genotyping array. Individuals were excluded on the basis of gender mismatches; minimal or excessive heterozygosity, disproportionate levels of individual missingness (>3%), insufficient sample replication (IBD <0.8), non-European ancestry (assessed by multidimensional scaling analysis and compared with Hapmap II) and cryptic relatedness (IBD > 0.1). SNPs were excluded based on minor allele frequency (<1%), call rate (<95%) or evidence for violations of Hardy-Weinberg equilibrium (P < 5E-7). Imputation was conducted by the ALSPAC team using Impute V2.2.2 against the 1000 genomes reference panel (Phase 1, Version 3: all polymorphic SNPs excluding singletons), using all 2186 reference haplotypes (including non-Europeans). SNPs were subsequently filtered based on minor allele frequency (<1%) and imputation quality (INFO<0.8). Following quality control and limiting individuals to one child per family, genetic data were available for N=7975.

Genome-wide association study (GWAS) were filtered to remove SNPs that were palindromic, insertions/deletions, non-autosomal, INFO score <0.8, missing in N>1 study and duplicates (https://github.com/ricanney). Depression results for 23andme (75,607 cases and 231,747 controls) (Hyde et al. 2016) and the other samples included in the latest depression GWAS (Wray et al. 2018) (PGC29, deCODE, Generation Scotland, GERA, iPSYCH, and UK Biobank) were meta-analysed in METAL.

PRS were generated for individuals in ALSPAC as the number of disorder risk alleles – defined using the GWAS summary statistics - weighted by effect size, using PRSice (Euesden et al. 2015); SNPs were clumped with an R^2^ threshold of 0.1 and a distance threshold of 1000kb and excluding the extended major histocompatibility complex (MHC; chromosome 6: 26-33Mb) due to the high linkage disequilibrium (LD) within this region. In the primary analyses we defined risk alleles as those associated with case-status at p<0.05 as this threshold has previously been shown to maximally capture phenotypic variance for schizophrenia (Schizophrenia Working Group of the Psychiatric Genomics Consortium 2014). Associations with factor scores for PRS generated using a range of different p-thresholds are shown in Supplementary Figure 1 and the number of SNPs included for each p-threshold given in Supplementary Table V. Polygenic risk scores were standardized using Z-score transformation. Correlations between the different PRS are also shown in Supplementary Table V.

**Identifying the best model fit**

We used confirmatory factor analysis to compare three models commonly used to examine hierarchically structured constructs (4-6): a correlated factors model (e.g. Supplementary Figures 2a), a bifactor model, (e.g. Supplementary Figure 2d), and a one factor model (Supplementary Figure 2e). Model fit was assessed using a variety of indices including sample size adjusted Bayesian information criteria, chi-square value, the comparative fit index (CFI, >0.95 considered good fit), Tucker-Lewis index (TFI, >0.95 considered good fit) and the root-mean-square error of approximation (RMSEA, <0.06 considered good fit) (7). Analyses were conducted in Mplus using a maximum likelihood parameter estimator for which standard errors are robust to non-normality (MLR) (8).

Model fit indices are presented in Supplementary Table VI and factor loadings in Supplementary Tables VII (age 7 years) and VII (age 13 years).

*Age 7 years*

Our initial, correlated factors model included three factors: emotional, behavioural and neurodevelopmental problems (Figure 2a). This model did not fit the data very well (Supplementary Table VI) and loading for the emotional factor were much lower for the depression and anxiety items than for irritability (see Supplementary Table VII), suggesting that irritability may not be best placed on this factor. Given these problems with model fit, and that it is not currently clear where irritability best fits within the emotional/behavioural/neurodevelopmental framework, we ran an exploratory analysis allowing irritability to load onto all three factors. The loadings of irritability onto the emotional and neurodevelopmental factors were small (standardized factor loading = 0.126, SE=0.015 p<0.001 and -0.135, SE=0.028, p<0.001), whereas irritability loaded well onto the behavioural factor (standardized factor loading = 0.884, SE=0.027, p<0.001). (Interestingly standard errors for loadings of irritability onto both the behavioural and neurodevelopmental factors were larger than for the other indicators.) In our amended three correlated factors model, we therefore included irritability as an indicator of behavioural problems (Figure 2b).

The amended correlated factors model fit the data reasonably well (see Supplementary Table VI), although the correlation between the behavioural and neurodevelopmental factors was high (r=0.766; see Supplementary Table VII). We therefore ran a two correlated factors model including one emotional and one behavioural/neurodevelopmental factor (Figure 2c). However, this model did not fit the data as well as the (amended) three correlated factors model (see Supplementary Table VI), suggesting a distinction between behavioural and neurodevelopmental problems. We therefore deemed the three-factor model the optimum model compared to the two-factor model.

In the next stage of model fitting, we ran a bifactor model by including a general psychopathology factor (Figure 2d), which fit the data well (see Supplementary Table VI); model indices suggested an improvement in fit compared to the correlated factor model. However, the conduct disorder item loaded negatively onto the behavioural factor (see Supplementary Table VII) – we therefore re-ran the model excluding conduct disorder from the specific behavioural factor (Figure 2e), which fit the data well (see Supplementary Table VI).

Reduced loading onto the specific factors in this model, compared to the correlated factor model, implies that these symptoms are more indicative of general psychopathology than the specific factor. This was most notable for the behavioural and neurodevelopmental problems. In contrast, loadings onto the emotional factor were less attenuated for the four anxiety measures, suggesting that these are more indicative of this specific factor than of general psychopathology.

Given previous work suggesting alternative bifactor models, for example with two specific emotional and behavioural factors, and autistic symptoms loading only onto the general psychopathology factor (9), we ran this alternative bifactor model (Figure 2f). However, factors loadings for ADHD symptoms onto a behavioural factor were negative (Supplementary Table VII), again suggesting a distinction between behavioural and neurodevelopmental problems.

In the final stage of model fitting, we ran a one factor model by removing the specific emotional, behavioural and neurodevelopmental factors (Supplementary Figure 2g). This model did not fit the data well (Supplementary Table VI), suggesting that a single general psychopathology factor cannot adequately model our measures of psychopathology.

The best fitting model was therefore a bifactor model which included a ‘general psychopathology’ factor as well as specific emotional, behavioural and neurodevelopmental factors (Figure 2e).

*Age 13 years*

For the age 13 data, we found a similar pattern of findings to age 7. Our initial, three correlated factors model again suggested that irritability may not be best placed on the emotional problems factor. ‘Exploratory’ analysis suggested that irritability was an indicator of behavioural problems (standardized factor loading = 0.927, SE=0.031, p<0.001), rather than emotional (standardized factor loading = 0.148, SE=0.016 p<0.001) or neurodevelopmental problems (standardized factor loading = -0.133, SE=0.035, p<0.001). (Again, standard errors for loadings of irritability onto both the behavioural and neurodevelopmental factors were larger than for the other indicators.) An amended three correlated factors model fit the data reasonably well, and better than the two correlated factors model. As at age 7, the bifactor model fit the data showed improvement in model fit compared to the correlated factor model; factor loadings were similar to age 7 and conduct disorder was removed from the behavioural factor. Again, in an alternative bifactor model ADHD symptoms loaded negatively onto the behavioural factor. Finally, a one factor model did not fit the data well. All model fit indices are shown in Supplementary Table VI and factor loadings in Supplementary Table VIII.

The best fitting model at age 13 years was therefore also a bifactor model which included a ‘general psychopathology’ factor and three specific factors of emotional, behavioural and neurodevelopmental problems (Figure 2e).

*Exploratory factor analyses*

In a final set of investigations, we conducted exploratory factor analyses. Eigenvalues suggested a three-factor solution (see Supplementary Figure 3), which fit the data well (see Supplementary Table IX for model fit indices for one-to-four factor solutions). We first modelled an exploratory three correlated factor model, which was consistent with there being an “emotional” factor (factor 1), a” behavioural” factor (factor 2) and a “neurodevelopmental” factor (factor 3) (Supplementary Table X). We then modelled an exploratory bifactor model, which showed a similar pattern of loadings to our final (confirmatory factor analysis) model for a “general psychopathology” factor (factor 1), an “emotional” factor (factor 2) and a “neurodevelopmental” factor (factor 3), but did not include a specific “behavioural” factor – instead the behavioural items were those that loaded most highly onto the “general psychopathology” factor (also in Supplementary Table X).

**Inverse probability weighting**

Inverse probability weighting (IPW) was used to assess the impact of missing genetic data and has been recommended over alternative methods such as multiple imputation in situations where whole blocks of data are missing for a large proportion of individuals (Seaman et al. 2012). Weights were derived from a logistic regression analysis of missing genetic data for those in the ‘core’ ALSPAC sample (N=6298/13793) for a set of measures assessed in pregnancy with minimal missingness: child gender (0% missing), child birth weight (1.3% missing: singly imputed) and maternal age (0% missing). Differences between those with and without genetic data for these variables are shown in Supplementary Table XI. Analyses conducted using IPW to address any potential bias caused by only a subsample having genetic data revealed a similar pattern of results (see Supplementary Table IV).

| **Supplementary Figure 1.** Multivariable associations between the bifactor model and polygenic risk scores of varying p-value thresholds | |
| --- | --- |
| a) Age 7 years | |
| 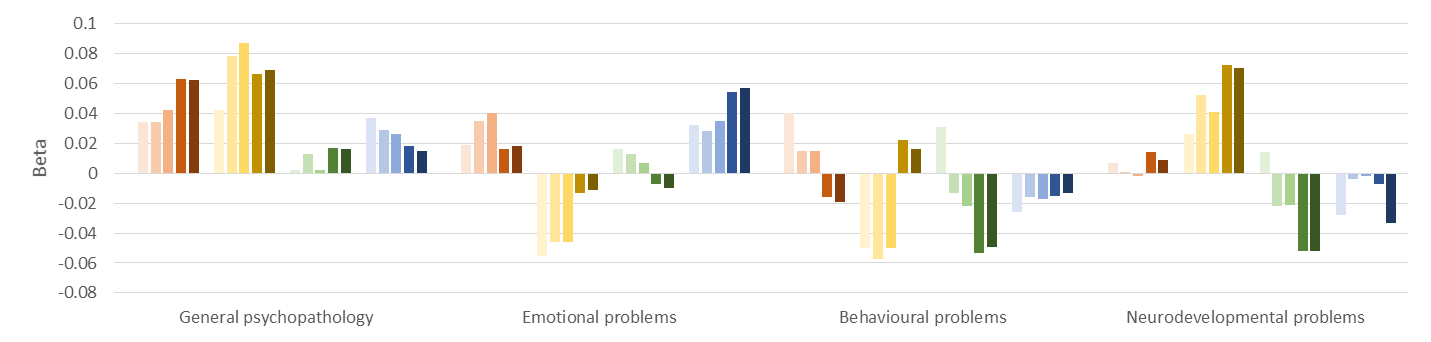 | |
| 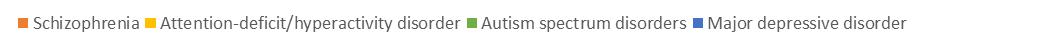 | 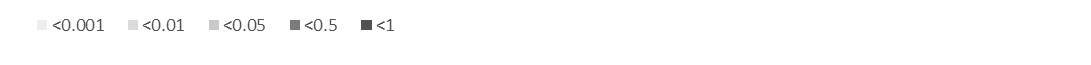 |
| b) Age 13 years | |
| 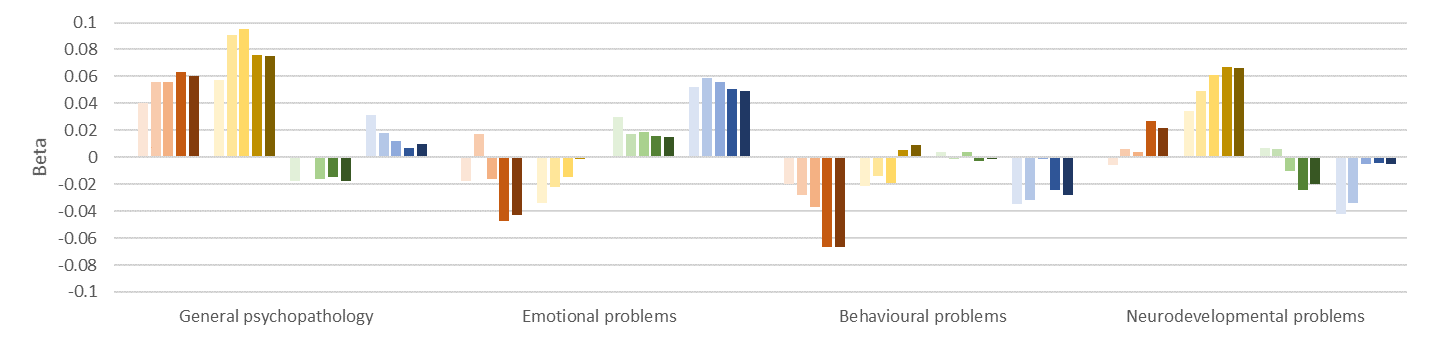 | |
| 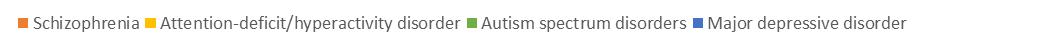 | 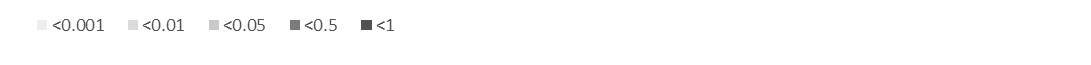 |

| **Supplementary Figure 2.** Factor models | | |
| --- | --- | --- |
| a) Initial three correlated factors model | b) Amended three correlated factors model | c) Two correlated factors model |
| 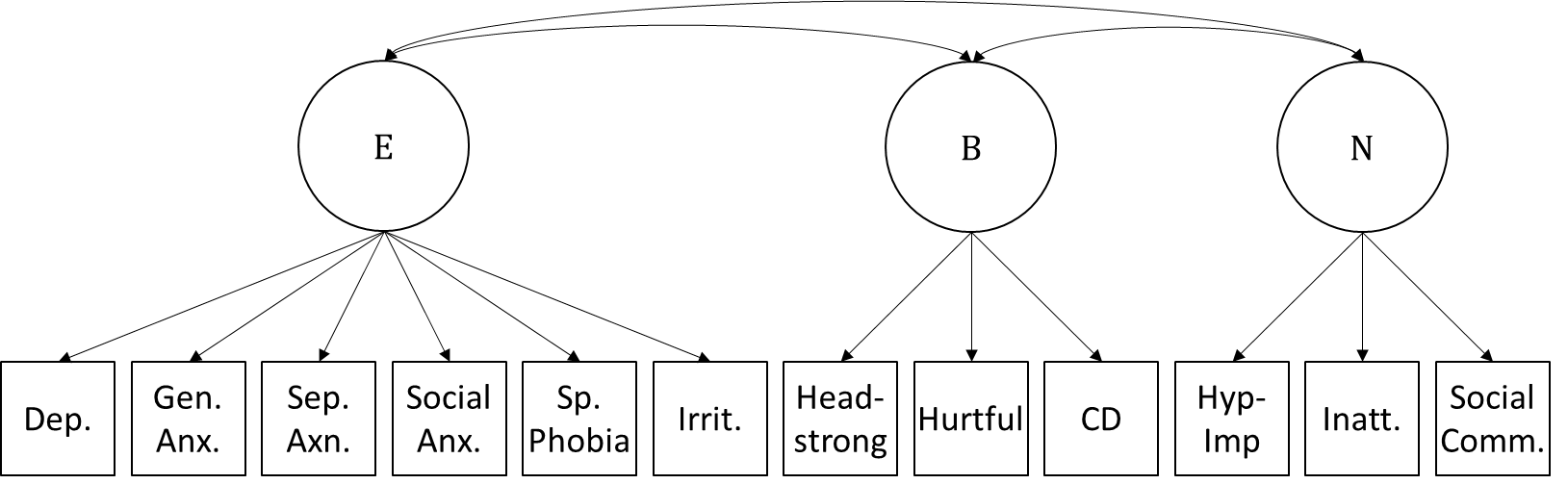 | 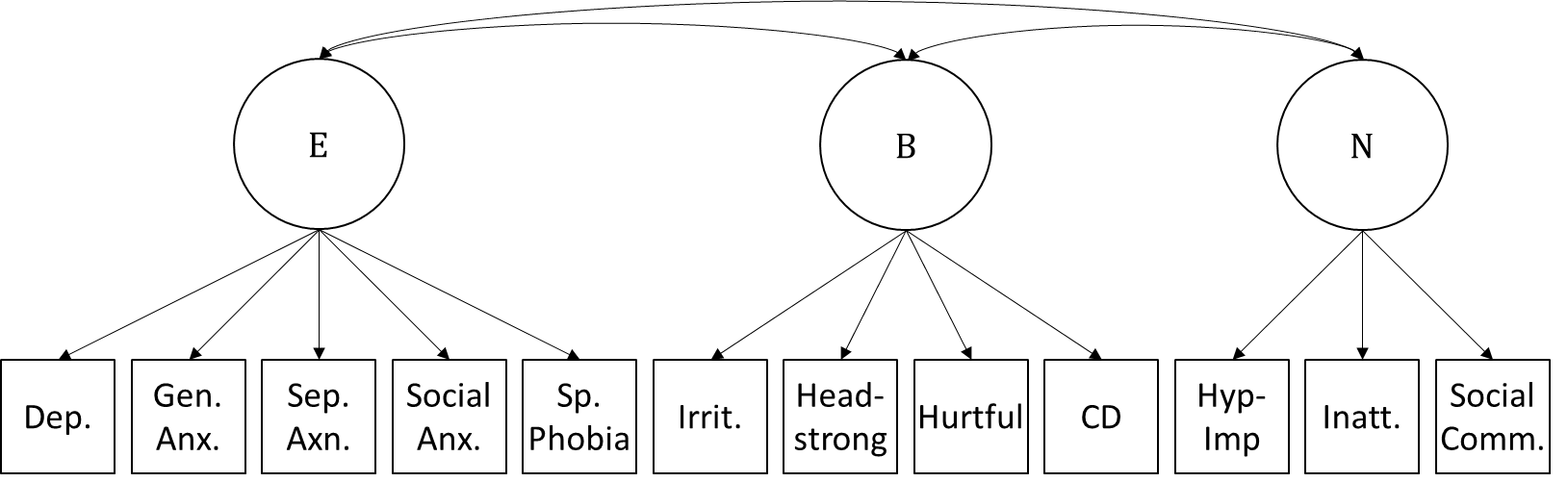 | 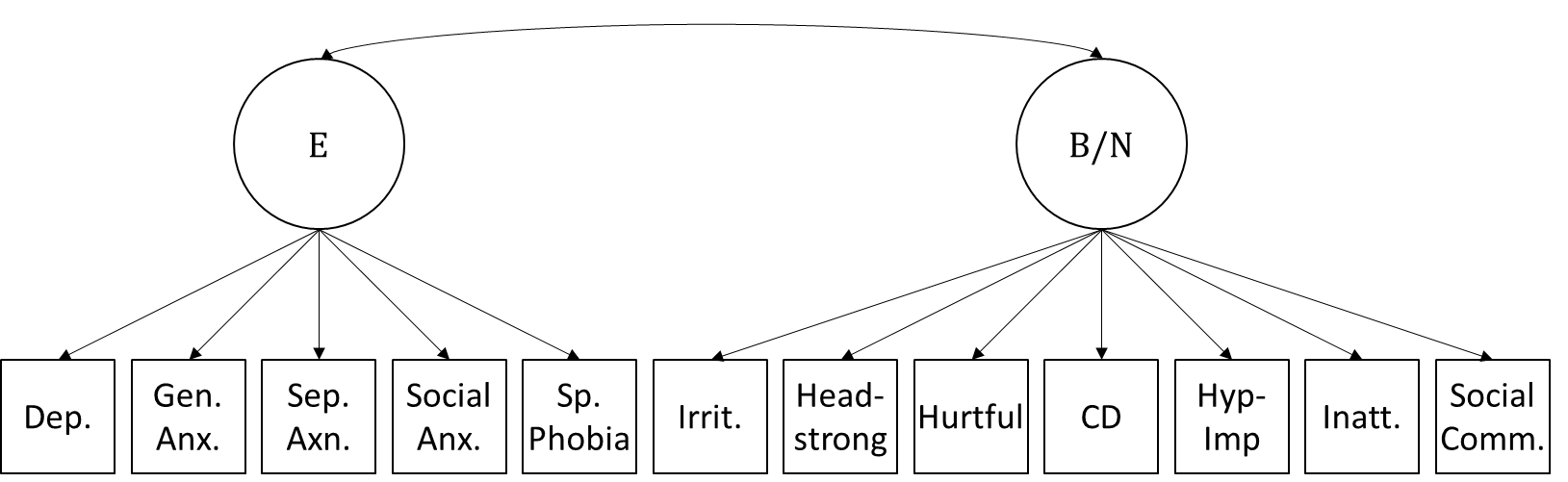 |
| d) Initial bifactor model | e) Final bifactor model | f) Alternative bifactor model |
| 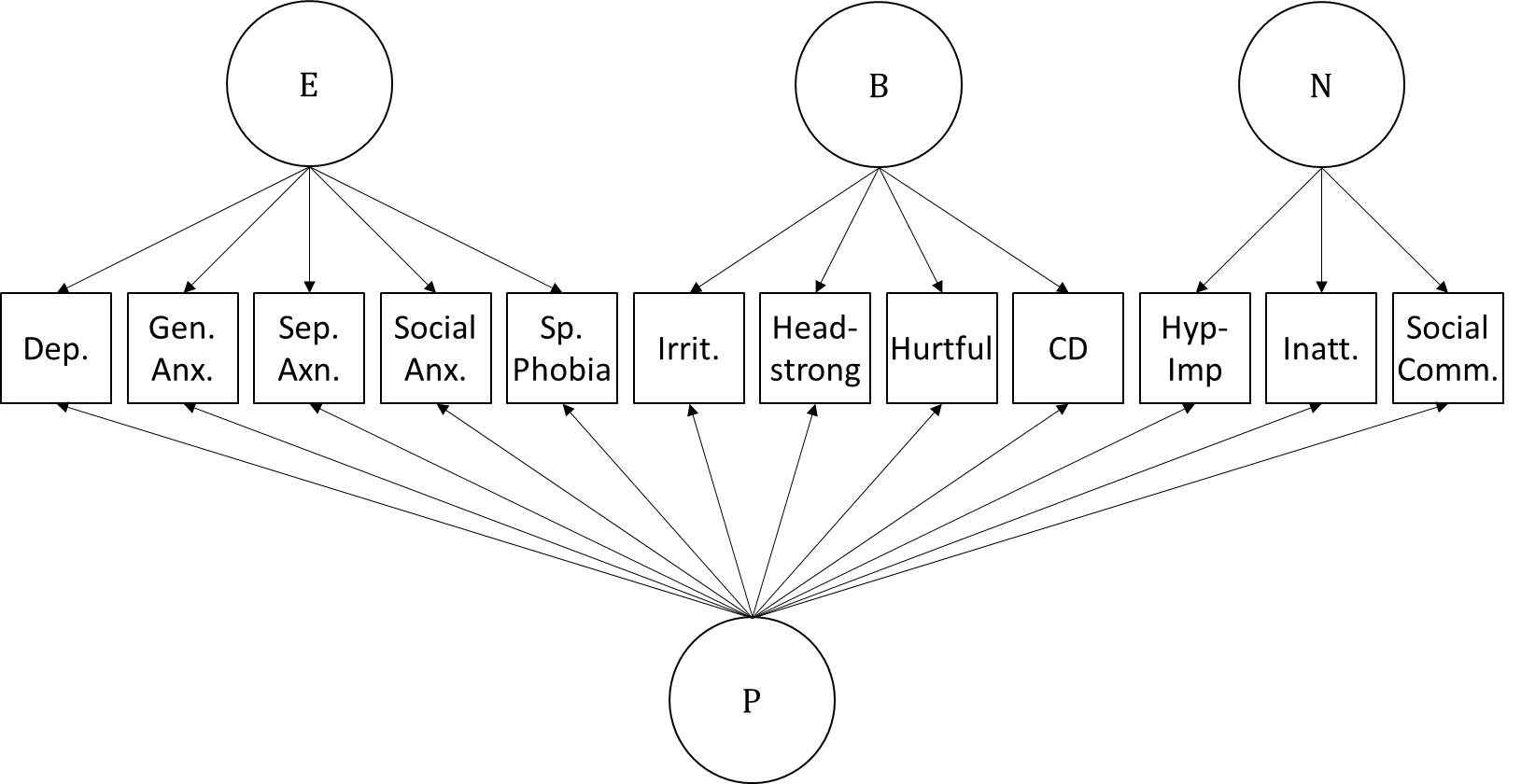 | 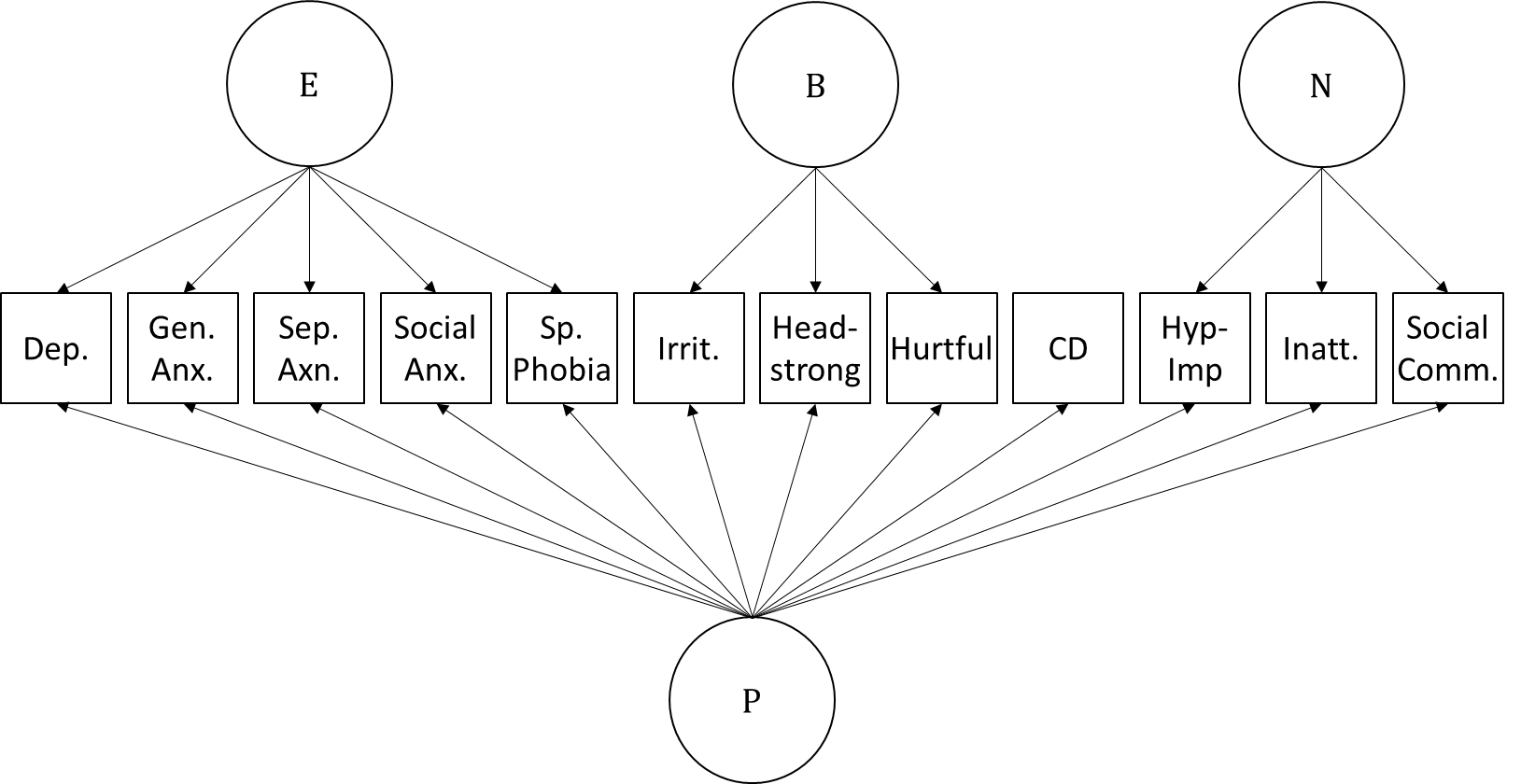 | 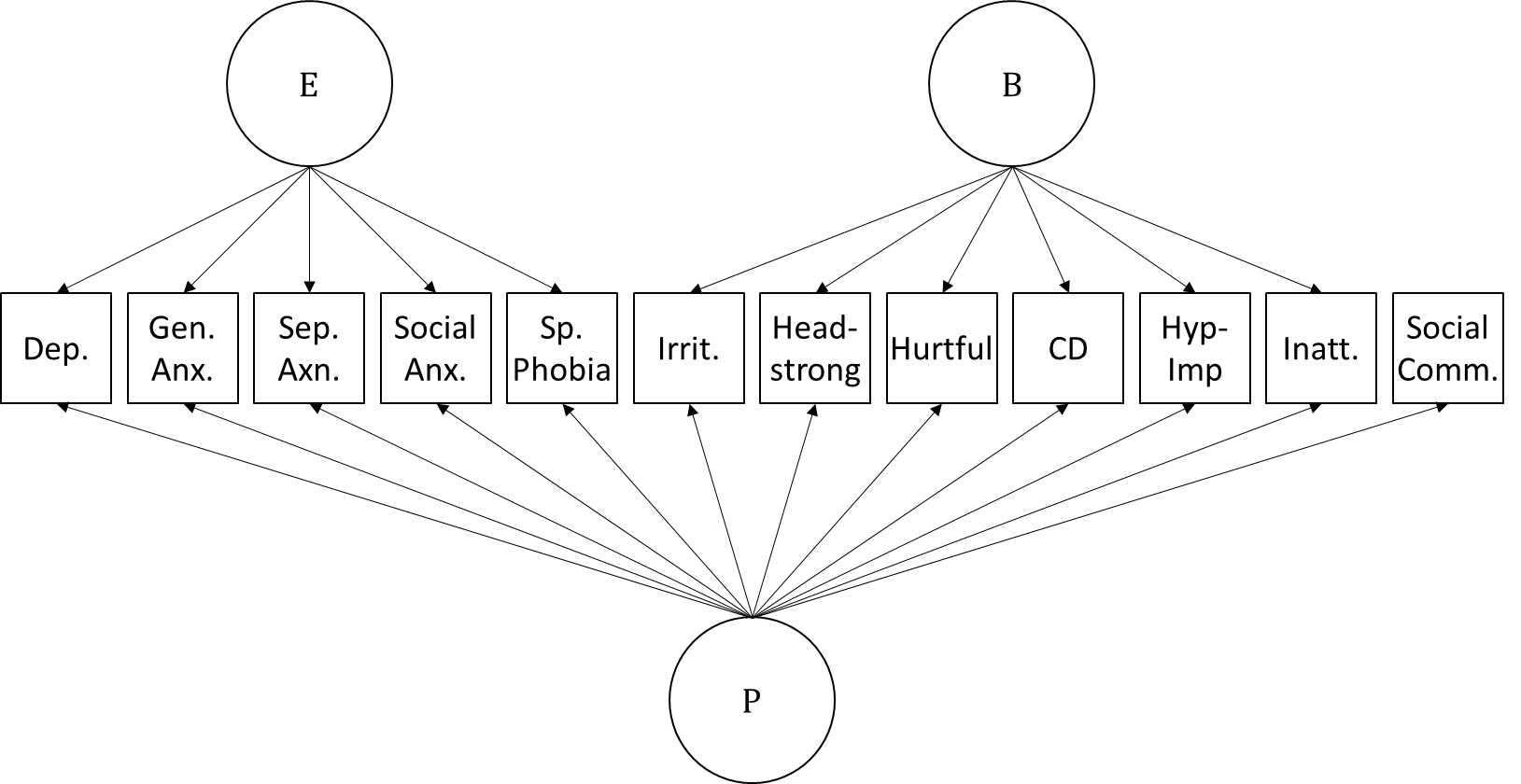 |
| g) One factor model |  |  |
| 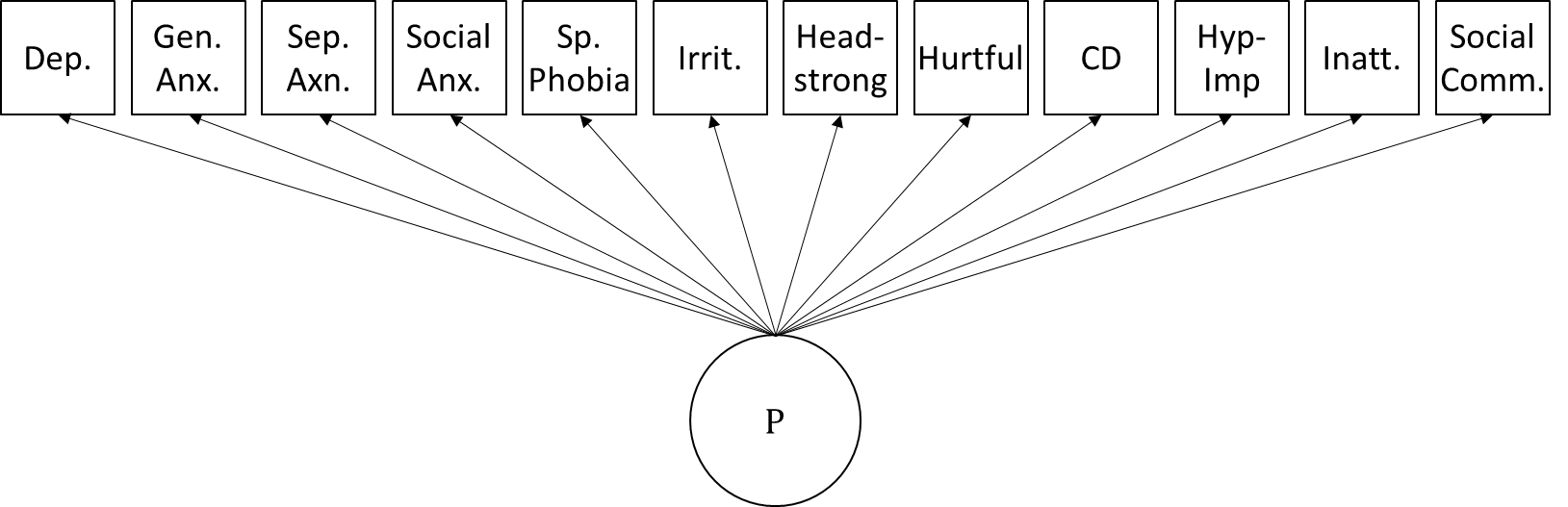 |  |  |
| E=emotional, B=behavioural, N=neurodevelopmental, P = general psychopathology, Dep=depression, Gen=Generalized, Anx=anxiety, Sep=separation, Sp=Specific, Irrit=irritability, CD=conduct disorder, Hyp-imp=Hyperactivity/impulsivity, Inatten=Inattentive, Comm=communication. | | |

| **Supplementary Figure 3.** Eigenvalues from exploratory factor analyses | |
| --- | --- |
| a) Age 7 years | b) Age 13 years |
| 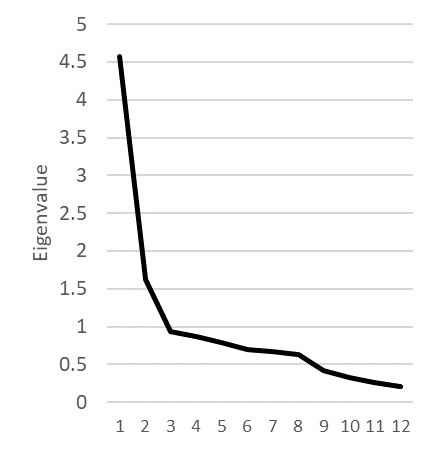 | 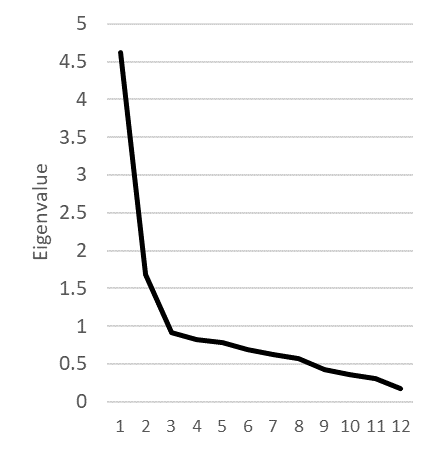 |

| **Supplementary Table I.** Descriptive statistics including correlations between variables at age 7 | | | | | | | | | | | | | | | | | | | | | | | | |  |
| --- | --- | --- | --- | --- | --- | --- | --- | --- | --- | --- | --- | --- | --- | --- | --- | --- | --- | --- | --- | --- | --- | --- | --- | --- | --- |
|  | 1. | | 2. | | 3. | | 4. | | 5. | | 6. | | 7. | | 8. | | 9. | | 10. | | 11. | | 12. | |  |
| 1. Depression | 1 | |  | |  | |  | |  | |  | |  | |  | |  | |  | |  | |  | |  |
| 1. Generalized anxiety | 0.280 | | 1 | |  | |  | |  | |  | |  | |  | |  | |  | |  | |  | |  |
| 1. Separation Anxiety | 0.267 | | 0.330 | | 1 | |  | |  | |  | |  | |  | |  | |  | |  | |  | |  |
| 1. Social anxiety | 0.165 | | 0.281 | | 0.250 | | 1 | |  | |  | |  | |  | |  | |  | |  | |  | |  |
| 1. Specific phobia | 0.111 | | 0.288 | | 0.297 | | 0.229 | | 1 | |  | |  | |  | |  | |  | |  | |  | |  |
| 1. Irritability | 0.278 | | 0.218 | | 0.255 | | 0.182 | | 0.134 | | 1 | |  | |  | |  | |  | |  | |  | |  |
| 1. Headstrong | 0.219 | | 0.170 | | 0.237 | | 0.145 | | 0.111 | | 0.758 | | 1 | |  | |  | |  | |  | |  | |  |
| 1. Hurtful | 0.198 | | 0.153 | | 0.201 | | 0.154 | | 0.105 | | 0.602 | | 0.644 | | 1 | |  | |  | |  | |  | |  |
| 1. Conduct disorder | 0.163 | | 0.131 | | 0.157 | | 0.078 | | 0.045 | | 0.380 | | 0.452 | | 0.418 | | 1 | |  | |  | |  | |  |
| 1. Hyperactivity-impulsivity | 0.188 | | 0.167 | | 0.208 | | 0.119 | | 0.116 | | 0.475 | | 0.593 | | 0.407 | | 0.395 | | 1 | |  | |  | |  |
| 1. Inattention | 0.217 | | 0.168 | | 0.196 | | 0.198 | | 0.115 | | 0.427 | | 0.499 | | 0.342 | | 0.346 | | 0.725 | | 1 | |  | |  |
| 1. Social-communication | 0.217 | | 0.167 | | 0.215 | | 0.185 | | 0.156 | | 0.589 | | 0.655 | | 0.472 | | 0.445 | | 0.661 | | 0.594 | | 1 | |  |
| Polygenic risk scores |  | |  | |  | |  | |  | |  | |  | |  | |  | |  | |  | |  | |  |
| Schizophrenia | -0.005 | | 0.022 | | 0.037 | | 0.064 | | 0.031 | | 0.048 | | 0.039 | | 0.026 | | 0.005 | | 0.025 | | 0.028 | | 0.053 | |  |
| Attention-deficit/hyperactivity disorder | 0.012 | | 0.002 | | 0.010 | | -0.019 | | -0.001 | | 0.029 | | 0.060 | | 0.048 | | 0.089 | | 0.093 | | 0.073 | | 0.058 | |  |
| Autism spectrum disorder | -0.007 | | 0.032 | | 0.005 | | -0.009 | | -0.011 | | 0.008 | | 0.003 | | 0.005 | | 0.001 | | 0.014 | | 0.003 | | 0.026 | |  |
| Major depressive disorder | 0.022 | | 0.045 | | 0.009 | | -0.003 | | 0.031 | | 0.020 | | 0.022 | | 0.009 | | 0.034 | | 0.029 | | 0.021 | | 0.028 | |  |
| N | 7927 | | 8084 | | 7911 | | 8030 | | 8146 | | 7961 | | 8026 | | 7969 | | 8015 | | 8027 | | 8015 | | 8015 | |  |
| Mean | 0.31 | | 1.42 | | 0.89 | | 0.89 | | 3.50 | | 0.49 | | 0.75 | | 0.15 | | 0.57 | | 2.47 | | 2.50 | | 2.84 | |  |
| (SD) | (1.02) | | (1.86) | | (2.01) | | (1.61) | | (2.52) | | (1.09) | | (1.53) | | (0.56) | | (1.05) | | (3.64) | | (3.72) | | (3.73) | |  |
| Cronbach's α | 0.78 | | 0.72 | | 0.81 | | 0.77 | | 0.59 | | 0.81 | | 0.86 | | 0.80 | | 0.51 | | 0.91 | | 0.92 | | 0.88 | |  |
| **Supplementary Table II.** Descriptive statistics including correlations between variables at age 13 years | | | | | | | | | | | | | | | | | | | | | | | | | |
|  | | 1. | | 2. | | 3. | | 4. | | 5. | | 6. | | 7. | | 8. | | 9. | | 10. | | 11. | | 12. | |
| 1. Depression | | 1 | |  | |  | |  | |  | |  | |  | |  | |  | |  | |  | |  | |
| 1. Generalized anxiety | | 0.305 | | 1 | |  | |  | |  | |  | |  | |  | |  | |  | |  | |  | |
| 1. Separation Anxiety | | 0.275 | | 0.338 | | 1 | |  | |  | |  | |  | |  | |  | |  | |  | |  | |
| 1. Social anxiety | | 0.206 | | 0.275 | | 0.207 | | 1 | |  | |  | |  | |  | |  | |  | |  | |  | |
| 1. Specific phobia | | 0.114 | | 0.357 | | 0.253 | | 0.249 | | 1 | |  | |  | |  | |  | |  | |  | |  | |
| 1. Irritability | | 0.338 | | 0.198 | | 0.241 | | 0.200 | | 0.100 | | 1 | |  | |  | |  | |  | |  | |  | |
| 1. Headstrong | | 0.278 | | 0.131 | | 0.210 | | 0.152 | | 0.050 | | 0.810 | | 1 | |  | |  | |  | |  | |  | |
| 1. Hurtful | | 0.234 | | 0.113 | | 0.190 | | 0.145 | | 0.061 | | 0.621 | | 0.642 | | 1 | |  | |  | |  | |  | |
| 1. Conduct disorder | | 0.256 | | 0.092 | | 0.124 | | 0.108 | | 0.016 | | 0.440 | | 0.502 | | 0.453 | | 1 | |  | |  | |  | |
| 1. Hyperactivity-impulsivity | | 0.223 | | 0.163 | | 0.203 | | 0.144 | | 0.068 | | 0.462 | | 0.525 | | 0.383 | | 0.343 | | 1 | |  | |  | |
| 1. Inattention | | 0.274 | | 0.170 | | 0.174 | | 0.230 | | 0.089 | | 0.471 | | 0.516 | | 0.345 | | 0.396 | | 0.664 | | 1 | |  | |
| 1. Social-communication | | 0.293 | | 0.184 | | 0.196 | | 0.213 | | 0.113 | | 0.618 | | 0.648 | | 0.483 | | 0.457 | | 0.573 | | 0.565 | | 1 | |
| Polygenic risk scores | |  | |  | |  | |  | |  | |  | |  | |  | |  | |  | |  | |  | |
| Schizophrenia | | 0.016 | | -0.004 | | 0.031 | | 0.025 | | -0.006 | | 0.031 | | 0.042 | | -0.004 | | 0.018 | | 0.020 | | 0.070 | | 0.051 | |
| Attention-deficit/hyperactivity disorder | | 0.037 | | -0.008 | | 0.054 | | 0.007 | | 0.028 | | 0.060 | | 0.075 | | 0.047 | | 0.081 | | 0.091 | | 0.091 | | 0.062 | |
| Autism spectrum disorder | | 0.022 | | 0.012 | | 0.027 | | 0.013 | | -0.001 | | -0.002 | | 0.016 | | -0.005 | | 0.004 | | 0.004 | | 0.022 | | -0.002 | |
| Major depressive disorder | | 0.051 | | 0.041 | | 0.028 | | 0.013 | | 0.026 | | 0.025 | | 0.015 | | 0.004 | | 0.015 | | 0.010 | | 0.034 | | 0.015 | |
| N | | 6825 | | 6910 | | 6340 | | 6908 | | 6969 | | 6870 | | 6901 | | 6844 | | 6922 | | 6939 | | 6921 | | 6932 | |
| Mean | | 0.42 | | 1.86 | | 0.49 | | 1.25 | | 3.70 | | 0.47 | | 0.61 | | 0.12 | | 0.60 | | 1.45 | | 2.65 | | 2.56 | |
| (SD) | | (1.32) | | (2.30) | | (1.47) | | (1.91) | | (3.03) | | (1.10) | | (1.43) | | (0.50) | | (1.31) | | (2.87) | | (3.95) | | (3.65) | |
| Cronbach's α | | 0.75 | | 0.61 | | 0.80 | | 0.79 | | 0.67 | | 0.85 | | 0.87 | | 0.79 | | 0.69 | | 0.90 | | 0.93 | | 0.89 | |

| **Supplementary Table III.** Univariable associations between genetic risk and the factor model at ages 7 and 13 years | | | | | | | | | | | | | | | | |
| --- | --- | --- | --- | --- | --- | --- | --- | --- | --- | --- | --- | --- | --- | --- | --- | --- |
| Age 7 | Schizophrenia PRS | | | | ADHD PRS | | | | ASD PRS | | | | Depression PRS | | | |
|  | β | SE | p | R^2^ | β | SE | p | R^2^ | β | SE | p | R^2^ | β | SE | p | R^2^ |
| General psychopathology | 0.048 | 0.018 | 0.006 | 0.002 | 0.093 | 0.019 | <0.001 | 0.009 | 0.026 | 0.018 | 0.136 | 0.001 | 0.041 | 0.017 | 0.019 | 0.002 |
| Emotional problems | 0.042 | 0.019 | 0.026 | 0.002 | -0.039 | 0.020 | 0.048 | 0.002 | 0.002 | 0.019 | 0.902 | <0.001 | 0.033 | 0.019 | 0.078 | 0.001 |
| Behavioural problems | 0.010 | 0.027 | 0.702 | <0.001 | -0.057 | 0.033 | 0.083 | 0.003 | -0.034 | 0.027 | 0.209 | 0.001 | -0.024 | 0.027 | 0.368 | 0.001 |
| Neurodevelopmental problems | -0.003 | 0.023 | 0.910 | <0.001 | 0.035 | 0.024 | 0.144 | 0.001 | -0.014 | 0.022 | 0.545 | <0.001 | 0.000 | 0.022 | 0.999 | <0.001 |
| Age 13 | Schizophrenia PRS | | | | ADHD PRS | | | | ASD PRS | | | | Depression PRS | | | |
|  | β | SE | p | R^2^ | β | SE | p | R^2^ | β | SE | p | R^2^ | β | SE | p | R^2^ |
| General psychopathology | 0.059 | 0.023 | 0.010 | 0.004 | 0.095 | 0.019 | <0.001 | 0.009 | 0.009 | 0.019 | 0.609 | <0.001 | 0.027 | 0.020 | 0.177 | 0.001 |
| Emotional problems | -0.010 | 0.020 | 0.626 | <0.001 | -0.004 | 0.019 | 0.828 | <0.001 | 0.021 | 0.018 | 0.250 | <0.001 | 0.055 | 0.018 | 0.003 | 0.003 |
| Behavioural problems | -0.033 | 0.036 | 0.316 | 0.001 | -0.019 | 0.030 | 0.529 | <0.001 | -0.001 | 0.031 | 0.965 | <0.001 | -0.005 | 0.031 | 0.860 | <0.001 |
| Neurodevelopmental problems | 0.006 | 0.041 | 0.887 | <0.001 | 0.059 | 0.024 | 0.012 | 0.009 | 0.005 | 0.025 | 0.846 | <0.001 | 0.003 | 0.028 | 0.925 | <0.001 |
| ADHD=attention-deficit/hyperactivity disorder, ASD=autism spectrum disorder, PRS=polygenic risk score | | | | | | | | | | | | | | | | |

| **Supplementary Table IV.** Multivariable associations between genetic risk and the factor model at age 7 years using inverse probability weighting | | | | | | | | | | | | | |
| --- | --- | --- | --- | --- | --- | --- | --- | --- | --- | --- | --- | --- | --- |
|  | Schizophrenia PRS | | | ADHD PRS | | | ASD PRS | | | MDD PRS | | |  |
|  | β | SE | p | β | SE | p | β | SE | p | β | SE | p | R^2^ |
| General psychopathology | 0.042 | 0.018 | 0.019 | 0.092 | 0.020 | <0.001 | -0.001 | 0.019 | 0.943 | 0.027 | 0.018 | 0.126 | 0.012 |
| Emotional problems | 0.041 | 0.020 | 0.036 | -0.045 | 0.021 | 0.031 | 0.007 | 0.019 | 0.729 | 0.033 | 0.020 | 0.094 | 0.005 |
| Behavioural problems | 0.025 | 0.029 | 0.388 | -0.053 | 0.037 | 0.147 | -0.024 | 0.030 | 0.422 | -0.014 | 0.029 | 0.618 | 0.005 |
| Neurodevelopmental problems | 0.000 | 0.024 | 0.995 | 0.040 | 0.026 | 0.131 | -0.019 | 0.024 | 0.421 | -0.001 | 0.022 | 0.961 | 0.002 |
| ASD=autism spectrum disorder, ADHD=attention-deficit/hyperactivity disorder, MDD=major depressive disorder, PRS=polygenic risk score | | | | | | | | | | | | | |

| **Supplementary Table V.** Correlations between and number of SNPs included in polygenic risk scores | | | | |
| --- | --- | --- | --- | --- |
| N=6166 | Schizophrenia | ADHD | ASD | Depression |
| Correlations^*^ |  |  |  |  |
| Schizophrenia | 1 |  |  |  |
| ADHD | 0.040^**^ | 1 |  |  |
| ASD | 0.052^**^ | 0.214^**^ | 1 |  |
| Depression | 0.086^**^ | 0.128^**^ | 0.109^**^ | 1 |
| Number of SNPs included |  |  |  |  |
| <0.001 | 4952 | 1459 | 1466 | 2951 |
| <0.01 | 16344 | 7020 | 8396 | 12829 |
| <0.05 | 40917 | 21278 | 27645 | 36266 |
| <0.5 | 144590 | 90683 | 126370 | 145440 |
| <1 | 183989 | 117699 | 164838 | 188085 |
| ADHD=attention-deficit/hyperactivity disorder, ASD=autism spectrum disorder, SNPs=single-nucleotide polymorphisms. ^*^p<0.01 ^**^p<0.001. | | | | |

| **Supplementary Table VI.** Model fit indices for confirmatory factor analyses | | | | | | | |
| --- | --- | --- | --- | --- | --- | --- | --- |
|  | Free parameters | ssaBIC | χ^2^ | df | CFI | TLI | RMSEA |
| Age 7 years, N=8161 |  |  |  |  |  |  |  |
| 1. Initial correlated factors model | 39 | 348286.628 | 2666.703 | 51 | 0.862 | 0.821 | 0.079 |
| 1. Amended correlated factors model | 39 | 345825.663 | 1262.224 | 51 | 0.936 | 0.917 | 0.054 |
| 1. Two correlated factors model | 37 | 348706.111 | 2915.369 | 53 | 0.849 | 0.812 | 0.081 |
| 1. Initial bifactor model | 48 | 344610.374 | 834.721 | 42 | 0.958 | 0.934 | 0.048 |
| 1. Final bifactor model | 47 | 344604.588 | 552.844 | 43 | 0.973 | 0.959 | 0.038 |
| 1. Alternative bifactor model | 47 | 344552.830 | 520.319 | 43 | 0.975 | 0.961 | 0.037 |
| 1. One factor model | 36 | 351279.050 | 4320.667 | 54 | 0.775 | 0.724 | 0.098 |
| Age 13 years, N=7017 |  |  |  |  |  |  |  |
| 1. Initial correlated factors model | 39 | 303661.957 | 2270.358 | 51 | 0.846 | 0.801 | 0.079 |
| 1. Amended correlated factors model | 39 | 301406.093 | 1138.158 | 51 | 0.925 | 0.903 | 0.055 |
| 1. Two correlated factors model | 37 | 303267.275 | 2069.647 | 53 | 0.861 | 0.826 | 0.074 |
| 1. Bifactor model | 48 | 300132.747 | 511.644 | 42 | 0.968 | 0.949 | 0.040 |
| 1. Final bifactor model | 47 | 300129.966 | 486.516 | 43 | 0.969 | 0.953 | 0.038 |
| 1. Alternative bifactor model | 47 | 300123.130 | 474.142 | 43 | 0.970 | 0.954 | 0.038 |
| 1. One factor model | 36 | 305435.370 | 3100.896 | 54 | 0.789 | 0.742 | 0.090 |
| ssaBIC = sample size adjusted Bayesian information criteria, df = degrees of freedom, CFI= comparative fit index, TLI = Tucker-Lewis index, RMSEA = root-mean-square error of approximation. Final model highlighted. | | | | | | | |

| **Supplementary Table VII.** Factor loadings and correlations at age 7 years (confirmatory factor analyses) | | | | | | | | | | | | | | | | | | | | | | | | | | | |
| --- | --- | --- | --- | --- | --- | --- | --- | --- | --- | --- | --- | --- | --- | --- | --- | --- | --- | --- | --- | --- | --- | --- | --- | --- | --- | --- | --- |
| Model | A | | | B | | | | C | | | D | | | | | | E | | | | | | F | | | | G |
| Loadings | E | B | N | E | B | | N | E | B/N | | E | B | | N | P | | E | B | | N | P | | E | B | | P | P |
| Dep. | 0.327 |  |  | 0.441 |  | |  | 0.443 |  | | 0.311 |  | |  | 0.292 | | 0.311 |  | |  | 0.292 | | 0.320 |  | | 0.279 | 0.307 |
| Gen. Anx. | 0.277 |  |  | 0.583 |  | |  | 0.582 |  | | 0.565 |  | |  | 0.229 | | 0.566 |  | |  | 0.230 | | 0.568 |  | | 0.223 | 0.256 |
| Sep. Anx. | 0.329 |  |  | 0.599 |  | |  | 0.598 |  | | 0.500 |  | |  | 0.296 | | 0.500 |  | |  | 0.296 | | 0.507 |  | | 0.282 | 0.315 |
| Social Anx. | 0.236 |  |  | 0.449 |  | |  | 0.450 |  | | 0.402 |  | |  | 0.206 | | 0.402 |  | |  | 0.206 | | 0.405 |  | | 0.201 | 0.231 |
| Sp. Phobia | 0.186 |  |  | 0.446 |  | |  | 0.445 |  | | 0.448 |  | |  | 0.157 | | 0.447 |  | |  | 0.158 | | 0.449 |  | | 0.153 | 0.181 |
| Irrit. | 0.891 |  |  |  | 0.824 | |  |  | 0.791 | |  | 0.412 | |  | 0.734 | |  | 0.416 | |  | 0.731 | |  | 0.399 | | 0.744 | 0.792 |
| Headstrong |  | 0.907 |  |  | 0.915 | |  |  | 0.868 | |  | 0.366 | |  | 0.827 | |  | 0.374 | |  | 0.824 | |  | 0.335 | | 0.835 | 0.861 |
| Hurtful |  | 0.709 |  |  | 0.708 | |  |  | 0.678 | |  | 0.340 | |  | 0.627 | |  | 0.351 | |  | 0.622 | |  | 0.357 | | 0.626 | 0.676 |
| CD |  | 0.517 |  |  | 0.512 | |  |  | 0.533 | |  | -0.013 | |  | 0.560 | |  |  | |  | 0.557 | |  | 0.086 | | 0.516 | 0.533 |
| Hyp-Imp |  |  | 0.860 |  |  | | 0.857 |  | 0.729 | |  |  | | 0.540 | 0.694 | |  |  | | 0.535 | 0.697 | |  | -0.316 | | 0.825 | 0.729 |
| Inatt. |  |  | 0.786 |  |  | | 0.787 |  | 0.656 | |  |  | | 0.566 | 0.605 | |  |  | | 0.564 | 0.608 | |  | -0.345 | | 0.746 | 0.659 |
| Social-Comm. |  |  | 0.799 |  |  | | 0.801 |  | 0.789 | |  |  | | 0.207 | 0.791 | |  |  | | 0.199 | 0.795 | |  |  | | 0.795 | 0.789 |
| Correlations | E | B | N | E | B | | N | E | B/N | | E | B | | N | P | | E | B | | N | P | | E | B | | P | P |
| E | 1 |  |  | 1 |  | |  | 1 |  | | 1 |  | |  |  | | 1 |  | |  |  | | 1 |  | |  | 1 |
| B | 0.917 | 1 |  | 0.424 | 1 | |  | 0.457 | 1 | | 0 | 1 | |  |  | | 0 | 1 | |  |  | | 0 | 1 | |  |  |
| N | 0.688 | 0.780 | 1 | 0.413 | 0.766 | | 1 |  |  | | 0 | 0 | | 1 |  | | 0 | 0 | | 1 |  | |  |  | |  |  |
| P |  |  |  |  |  | |  |  |  | | 0 | 0 | | 0 | 1 | | 0 | 0 | | 0 | 1 | | 0 | 0 | | 1 |  |
| E=emotional, B=behavioural, N=neurodevelopmental, P = general psychopathology, Dep=depression, Gen=Generalized, Anx=anxiety, Sep=separation, Sp=Specific, Irrit=irritability, CD=conduct disorder, Hyp-imp=Hyperactivity/impulsivity, Inatten=Inattentive, Comm=communication. Final model = model E. | | | | | | | | | | | | | | | | | | | | | | | | | | | |
| **Supplementary Table VIII.** Factor loadings and correlations at age 13 years (confirmatory factor analyses) | | | | | | | | | | | | | | | | | | | | | | | | | | | |
| Model | A | | | B | | | | C | | | D | | | | | | E | | | | | | F | | | | G |
| Loadings | E | B | N | E | B | | N | E | B/N | | E | B | | N | P | | E | B | | N | P | | E | B | | P | P |
| Dep. | 0.384 |  |  | 0.499 |  | |  | 0.501 |  | | 0.303 |  | |  | 0.394 | | 0.306 |  | |  | 0.393 | | 0.316 |  | | 0.370 | 0.384 |
| Gen. Anx. | 0.233 |  |  | 0.630 |  | |  | 0.630 |  | | 0.663 |  | |  | 0.219 | | 0.664 |  | |  | 0.213 | | 0.664 |  | | 0.211 | 0.228 |
| Sep. Anx. | 0.292 |  |  | 0.535 |  | |  | 0.540 |  | | 0.435 |  | |  | 0.270 | | 0.436 |  | |  | 0.269 | | 0.440 |  | | 0.263 | 0.290 |
| Social Anx. | 0.236 |  |  | 0.455 |  | |  | 0.450 |  | | 0.362 |  | |  | 0.248 | | 0.365 |  | |  | 0.241 | | 0.367 |  | | 0.239 | 0.244 |
| Sp. Phobia | 0.125 |  |  | 0.446 |  | |  | 0.445 |  | | 0.498 |  | |  | 0.105 | | 0.500 |  | |  | 0.099 | | 0.498 |  | | 0.100 | 0.120 |
| Irrit. | 0.930 |  |  |  | 0.871 | |  |  | 0.859 | |  | 0.445 | |  | 0.760 | |  | 0.424 | |  | 0.777 | |  | 0.371 | | 0.804 | 0.858 |
| Headstrong |  | 0.919 |  |  | 0.924 | |  |  | 0.899 | |  | 0.447 | |  | 0.803 | |  | 0.399 | |  | 0.824 | |  | 0.329 | | 0.851 | 0.889 |
| Hurtful |  | 0.703 |  |  | 0.702 | |  |  | 0.695 | |  | 0.349 | |  | 0.610 | |  | 0.296 | |  | 0.636 | |  | 0.314 | | 0.636 | 0.693 |
| CD |  | 0.557 |  |  | 0.553 | |  |  | 0.566 | |  | 0.075 | |  | 0.579 | |  |  | |  | 0.601 | |  | 0.085 | | 0.556 | 0.568 |
| Hyp-Imp |  |  | 0.763 |  |  | | 0.761 |  | 0.628 | |  |  | | 0.620 | 0.633 | |  |  | | 0.596 | 0.616 | |  | -0.310 | | 0.733 | 0.632 |
| Inatt. |  |  | 0.758 |  |  | | 0.758 |  | 0.625 | |  |  | | 0.410 | 0.650 | |  |  | | 0.464 | 0.632 | |  | -0.366 | | 0.752 | 0.633 |
| Social-Comm. |  |  | 0.800 |  |  | | 0.801 |  | 0.753 | |  |  | | 0.109 | 0.804 | |  |  | | 0.157 | 0.784 | |  |  | | 0.771 | 0.757 |
| Correlations | E | B | N | E | B | | N | E | B/N | | E | B | | N | P | | E | B | | N | P | | E | B | | P | P |
| E | 1 |  |  | 1 |  | |  | 1 |  | | 1 |  | |  |  | | 1 |  | |  |  | | 1 |  | |  | 1 |
| B | 0.933 | 1 |  | 0.391 | 1 | |  | 0.433 | 1 | | 0 | 1 | |  |  | | 0 | 1 | |  |  | | 0 | 1 | |  |  |
| N | 0.743 | 0.806 | 1 | 0.455 | 0.795 | | 1 |  |  | | 0 | 0 | | 1 |  | | 0 | 0 | | 1 |  | |  |  | |  |  |
| P |  |  |  |  |  | |  |  |  | | 0 | 0 | | 0 | 1 | | 0 | 0 | | 0 | 1 | | 0 | 0 | | 1 |  |
| E=emotional, B=behavioural, N=neurodevelopmental, P = general psychopathology, Dep=depression, Gen=Generalized, Anx=anxiety, Sep=separation, Sp=Specific, Irrit=irritability, CD=conduct disorder, Hyp-imp=Hyperactivity/impulsivity, Inatten=Inattentive, Comm=communication. Final model = model E. | | | | | | | | | | | | | | | | | | | | | | | | | | | |
| **Supplementary Table IX.** Model fit indices for exploratory factor analyses | | | | | | | | | | | | | | | | | | | | | | | | | | | |
|  | | | | | | Free parameters | | | | ssaBIC | | | χ^2^ | | | df | | | CFI | | | TLI | | | RMSEA | | |
| Age 7 years, N=8161 | | | | | |  | | | |  | | |  | | |  | | |  | | |  | | |  | | |
| One factor | | | | | | 36 | | | | 351279.050 | | | 4320.703 | | | 54 | | | 0.775 | | | 0.724 | | | 0.098 | | |
| Two factors | | | | | | 47 | | | | 348483.667 | | | 3241.774 | | | 43 | | | 0.831 | | | 0.741 | | | 0.095 | | |
| Three factors | | | | | | 57 | | | | 344363.233 | | | 379.197 | | | 33 | | | 0.982 | | | 0.963 | | | 0.036 | | |
| Four factors | | | | | | 66 | | | | 344232.364 | | | 475.140 | | | 24 | | | 0.976 | | | 0.934 | | | 0.048 | | |
| Age 13 years, N=7017 | | | | | |  | | | |  | | |  | | |  | | |  | | |  | | |  | | |
| One factor | | | | | | 36 | | | | 305435.370 | | | 3100.849 | | | 54 | | | 0.789 | | | 0.742 | | | 0.090 | | |
| Two factors | | | | | | 47 | | | | 302461.865 | | | 1669.475 | | | 43 | | | 0.888 | | | 0.827 | | | 0.073 | | |
| Three factors | | | | | | 57 | | | | 299926.123 | | | 341.137 | | | 33 | | | 0.979 | | | 0.957 | | | 0.036 | | |
| Four factors | | | | | | 66 | | | | 299731.947 | | | 245.392 | | | 24 | | | 0.985 | | | 0.958 | | | 0.036 | | |
| ssaBIC = sample size adjusted Bayesian information criteria, df = degrees of freedom, CFI= comparative fit index, TLI = Tucker-Lewis index, RMSEA = root-mean-square error of approximation. Final model highlighted. | | | | | | | | | | | | | | | | | | | | | | | | | | | |

| **Supplementary Table X.** Factor loadings and correlations for exploratory factor analysis models | | | | | | | | | | | | |  |  |  |
| --- | --- | --- | --- | --- | --- | --- | --- | --- | --- | --- | --- | --- | --- | --- | --- |
| Model | Age 7: correlated factors | | | Age 7: Bifactor | | | Age 13: correlated factors | | | Age 13: Bifactor | | |  |  |  |
| Loadings | F1 | F2 | F3 | F1 | F2 | F3 | F1 | F2 | F3 | F1 | F2 | F3 |  |  |  |
| Dep. | 0.364 | 0.120 | 0.029 | 0.301 | 0.313 | 0.002 | 0.351 | 0.193 | 0.088 | 0.382 | 0.311 | 0.008 |  |  |  |
| Gen. Anx. | 0.613 | -0.008 | -0.016 | 0.253 | 0.549 | -0.002 | 0.689 | -0.007 | -0.001 | 0.236 | 0.645 | 0.010 |  |  |  |
| Sep. Anx. | 0.544 | 0.072 | 0.003 | 0.315 | 0.479 | -0.004 | 0.466 | 0.123 | 0.003 | 0.286 | 0.424 | -0.023 |  |  |  |
| Social Anx. | 0.457 | 0.010 | 0.009 | 0.220 | 0.408 | 0.009 | 0.396 | 0.012 | 0.113 | 0.244 | 0.372 | 0.067 |  |  |  |
| Sp. Phobia | 0.483 | -0.043 | -0.002 | 0.172 | 0.437 | 0.011 | 0.524 | -0.039 | -0.033 | 0.120 | 0.493 | -0.003 |  |  |  |
| Irrit. | 0.121 | 0.800 | -0.002 | 0.835 | 0.011 | -0.133 | 0.099 | 0.864 | -0.011 | 0.859 | 0.006 | -0.217 |  |  |  |
| Headstrong | -0.022 | 0.810 | 0.154 | 0.900 | -0.109 | -0.036 | -0.040 | 0.867 | 0.095 | 0.899 | -0.122 | -0.159 |  |  |  |
| Hurtful | 0.052 | 0.706 | -0.011 | 0.706 | -0.039 | -0.124 | 0.020 | 0.711 | -0.015 | 0.681 | -0.052 | -0.183 |  |  |  |
| CD | -0.001 | 0.343 | 0.232 | 0.513 | -0.029 | 0.092 | -0.024 | 0.403 | 0.220 | 0.559 | -0.057 | 0.027 |  |  |  |
| Hyp-Imp | -0.081 | 0.011 | 0.916 | 0.673 | -0.023 | 0.588 | -0.027 | 0.032 | 0.783 | 0.661 | -0.010 | 0.443 |  |  |  |
| Inatt. | 0.007 | -0.063 | 0.851 | 0.590 | 0.061 | 0.559 | 0.004 | -0.031 | 0.855 | 0.670 | 0.027 | 0.500 |  |  |  |
| Social-Comm. | 0.015 | 0.347 | 0.520 | 0.743 | 0.001 | 0.278 | 0.050 | 0.405 | 0.427 | 0.756 | 0.017 | 0.147 |  |  |  |
| Correlations | F1 | F2 | F3 | F1 | F2 | F3 | F1 | F2 | F3 | F1 | F2 | F3 |  |  |  |
| F1 | 1 |  |  | 1 |  |  | 1 |  |  | 1 |  |  |  |  |  |
| F2 | 0.326 | 1 |  | 0 | 1 |  | 0.245 | 1 |  | 0 | 1 |  |  |  |  |
| F3 | 0.396 | 0.632 | 1 | 0 | 0 | 1 | 0.318 | 0.644 | 1 | 0 | 0 | 1 |  |  |  |
| F1-F3=factors 1-3, Dep=depression, Gen=Generalized, Anx=anxiety, Sep=separation, Sp=Specific, Irrit=irritability, CD=conduct disorder, Hyp-imp=Hyperactivity/impulsivity, Inatten=Inattentive, Comm=communication. | | | | | | | | | | | | |  |  |  |

| **Supplementary Table XI.** Differences between those with and without polygenic risk score data on variables included in the inverse probability weighting model | | | |
| --- | --- | --- | --- |
|  | With PRS data (N=7495) | Without PRS data (N=6298) | Association with missingness |
| Child gender, female | 48.317% | 48.472% | OR=0.99 (0.93-1.06), p=0.856 |
| Child birth weight | Mean=3437.326 (SD=532.572) | Mean=3361.634 (SD=568.202) | OR=1.00 (1.00-1.00)^*^, p<0.001 |
| Maternal age | Mean=28.700 (SD=4.755) | Mean=27.119 (SD=5.086) | OR=0.94 (0.93-0.94), p<0.001 |
| PRS=polygenic risk score. N=13793 for maternal age and child gender, N=13619 for child birth weight. ^*^OR=0.9997 (0.9997-0.9998) | | | |

**References**

Hyde CL, Nagle MW, Tian C, Chen X, Paciga SA, Wendland JR, Tung JY, Hinds DA, Perlis RH, Winslow AR (2016) Identification of 15 genetic loci associated with risk of major depression in individuals of European descent. Nat Genet 48(9):1031-1036

Wray NR, Ripke S, Mattheisen M, Trzaskowski M, Byrne EM, Abdellaoui A, Adams MJ, Agerbo E, Air TM, Andlauer TMF, Bacanu SA, Baekvad-Hansen M, Beekman AFT, Bigdeli TB, Binder EB, Blackwood DRH, Bryois J, Buttenschon HN, Bybjerg-Grauholm J, Cai N, Castelao E, Christensen JH, Clarke TK, Coleman JIR, Colodro-Conde L, Couvy-Duchesne B, Craddock N, Crawford GE, Crowley CA, Dashti HS, Davies G, Deary IJ, Degenhardt F, Derks EM, Direk N, Dolan CV, Dunn EC, Eley TC, Eriksson N, Escott-Price V, Kiadeh FHF, Finucane HK, Forstner AJ, Frank J, Gaspar HA, Gill M, Giusti-Rodriguez P, Goes FS, Gordon SD, Grove J, Hall LS, Hannon E, Hansen CS, Hansen TF, Herms S, Hickie IB, Hoffmann P, Homuth G, Horn C, Hottenga JJ, Hougaard DM, Hu M, Hyde CL, Ising M, Jansen R, Jin F, Jorgenson E, Knowles JA, Kohane IS, Kraft J, Kretzschmar WW, Krogh J, Kutalik Z, Lane JM, Li Y, Li Y, Lind PA, Liu X, Lu L, MacIntyre DJ, MacKinnon DF, Maier RM, Maier W, Marchini J, Mbarek H, McGrath P, McGuffin P, Medland SE, Mehta D, Middeldorp CM, Mihailov E, Milaneschi Y, Milani L, Mill J, Mondimore FM, Montgomery GW, Mostafavi S, Mullins N, Nauck M, Ng B, Nivard MG, Nyholt DR, O'Reilly PF, Oskarsson H, Owen MJ, Painter JN, Pedersen CB, Pedersen MG, Peterson RE, Pettersson E, Peyrot WJ, Pistis G, Posthuma D, Purcell SM, Quiroz JA, Qvist P, Rice JP, Riley BP, Rivera M, Saeed Mirza S, Saxena R, Schoevers R, Schulte EC, Shen L, Shi J, Shyn SI, Sigurdsson E, Sinnamon GBC, Smit JH, Smith DJ, Stefansson H, Steinberg S, Stockmeier CA, Streit F, Strohmaier J, Tansey KE, Teismann H, Teumer A, Thompson W, Thomson PA, Thorgeirsson TE, Tian C, Traylor M, Treutlein J, Trubetskoy V, Uitterlinden AG, Umbricht D, Van der Auwera S, van Hemert AM, Viktorin A, Visscher PM, Wang Y, Webb BT, Weinsheimer SM, Wellmann J, Willemsen G, Witt SH, Wu Y, Xi HS, Yang J, Zhang F, eQtlgen, andMe, Arolt V, Baune BT, Berger K, Boomsma DI, Cichon S, Dannlowski U, de Geus ECJ, DePaulo JR, Domenici E, Domschke K, Esko T, Grabe HJ, Hamilton SP, Hayward C, Heath AC, Hinds DA, Kendler KS, Kloiber S, Lewis G, Li QS, Lucae S, Madden PFA, Magnusson PK, Martin NG, McIntosh AM, Metspalu A, Mors O, Mortensen PB, Muller-Myhsok B, Nordentoft M, Nothen MM, O'Donovan MC, Paciga SA, Pedersen NL, Penninx B, Perlis RH, Porteous DJ, Potash JB, Preisig M, Rietschel M, Schaefer C, Schulze TG, Smoller JW, Stefansson K, Tiemeier H, Uher R, Volzke H, Weissman MM, Werge T, Winslow AR, Lewis CM, Levinson DF, Breen G, Borglum AD, Sullivan PF, Major Depressive Disorder Working Group of the Psychiatric Genomics C (2018) Genome-wide association analyses identify 44 risk variants and refine the genetic architecture of major depression. Nat Genet 50(5):668-681

Euesden J, Lewis CM, O'Reilly PF (2015) PRSice: Polygenic Risk Score software. Bioinformatics 31(9):1466-1468

Seaman SR, White IR, Copas AJ, Li L (2012) Combining multiple imputation and inverse-probability weighting. Biometrics 68(1):129-137
